# Host genetics play a critical role in controlling CD8 T cell function and lethal immunopathology during chronic viral infection

**DOI:** 10.1371/journal.ppat.1006498

**Published:** 2017-07-17

**Authors:** Allison F. Christiaansen, Megan E. Schmidt, Stacey M. Hartwig, Steven M. Varga

**Affiliations:** 1 Department of Microbiology, University of Iowa, Iowa City, IA, United States of America; 2 Interdisciplinary Graduate Program in Immunology, University of Iowa, Iowa City, IA, United States of America; 3 Department of Pathology, University of Iowa, Iowa City, IA, United States of America; St. Jude Children's Research Hospital, UNITED STATES

## Abstract

Effective CD8 T cell responses are vital for the control of chronic viral infections. Many factors of the host immune response contribute to the maintenance of effector CD8 T cell responses versus CD8 T cell exhaustion during chronic infection. Specific MHC alleles and the degree of MHC heterogeneity are associated with enhanced CD8 T cell function and viral control during human chronic infection. However, it is currently unclear to what extent host genetics influences the establishment of chronic viral infection. In order to examine the impact of MHC heterogeneity versus non-MHC host genetics on the development of chronic viral infection, an F1 cross of B10.D2 x B6 (D2B6F1) and BALB.B x BALB/c (BCF1) mice were infected with the clone-13 (Cl-13) strain of lymphocytic choriomeningitis virus (LCMV). Following chronic Cl-13 infection both H-2^bxd^ D2B6F1 and BCF1 mice demonstrated increased viral control compared to homozygous mice. Strikingly, H-2^bxd^ D2B6F1 mice on a C57BL genetic background exhibited mortality following Cl-13 infection. CD8 T cell depletion prevented mortality in Cl-13-infected D2B6F1 mice indicating that mortality was CD8 T-cell-dependent. D2B6F1 mice maintained more CD8 T cell effector cytokine production and exhibited reduced expression of the T cell exhaustion marker PD-1. In addition, D2B6F1 mice also induced a larger Th1 response than BCF1 mice and Cl-13-induced mortality in D2B6F1 mice was also dependent on CD4 T-cell-mediated IFN-γ production. Thus, following a chronic viral infection, increased functionality of the CD8 T cell response allowed for more rapid viral clearance at the cost of enhanced immunopathology dependent on both MHC diversity and the genetic background of the host.

## Author summary

Chronic viral infections pose a serious healthcare concern resulting in substantial mortality worldwide. Chronic viral infections result from the inability of the immune system to eliminate the virus from the infected individual. The immune system’s inability to eradicate the invading pathogen is partially due to excessive regulation of the T cell response. However, host genetics have been associated with enhanced T cell function and viral control during chronic infection. Therefore, we sought to investigate the role of host genetic diversity on the T cell response during chronic viral infection in a murine model. We found that increasing MHC heterogeneity resulted in an increased T cell response and enhanced viral control. In addition, host genetic background differences allowed for induction of a distinct CD4 T cell subset, which was associated with reduced suppression of the CD8 T cell response and enhanced viral control. Thus, specific features of the host genetic background contribute to the size and quality of the T cell response and resulting viral control. This study identifies components of the T cell response that may provide a therapeutic target to enhance T-cell-mediated viral control during chronic infection.

## Introduction

Chronic viral infection such as human immunodeficiency virus (HIV) and hepatitis C virus (HCV) affect an estimated 185 million people worldwide. In the course of these infections HIV and HCV are able to evade the host immune response and establish viral persistence. Genetic factors that influence the host T cell response have been shown to greatly impact viral load and disease progression [1–4]. Expression of either heterozygous human leukocyte antigen (HLA) class I alleles or the specific HLA alleles HLA B*5701 and HLA B*2701 are associated with enhanced CD8 T cell responses and reduced viral loads during HIV infection [3, 5–8]. Clearance of HCV infection has also been associated with the expression of heterozygous HLA alleles as well as with the expression of specific HLA alleles [2, 4]. These host advantages driven by HLA expression patterns demonstrate the critical importance of the T cell response during chronic viral infection. In both humans and animal models of chronic viral infection, the capacity to maintain a functional CD8 T cell response has been shown to play a critical role in limiting viral replication [9–12]. However, constant CD8 T cell stimulation during chronic viral infection can drive CD8 T cells into a state of exhaustion where antiviral CD8 T cells progressively lose their effector functions, preventing effective clearance of infection [13–16]. CD8 T cell exhaustion is believed to occur in order to limit immunopathology to the host.

In addition to CD8 T cells, CD4 T cells also play a crucial role during the development of chronic viral infections. During HIV and murine infection with the persistent clone-13 (Cl-13) strain of lymphocytic choriomeningitis virus (LCMV), depletion of CD4 T cells is associated with reduced CD8 T cell effector functions and increased viral titers [11, 17]. Similar to CD8 T cells, CD4 T cell responses are altered by the presence of persistent antigen. Chronic viral infection drives CD4 T cell conversion from a Th1 phenotype critical for viral clearance to a T follicular helper (Tfh) phenotype [18, 19]. Thus, maintaining antiviral CD4 and CD8 T cell effector functions is vital to the control of chronic viral infection.

Murine models have often been used to delineate the contribution of MHC alleles versus host genetic background on mouse strain susceptibility to infection. BALB/c and C57BL/6 (B6) mice have been shown to differ in their susceptibility to infections in both MHC-dependent [20–22] and -independent manners [23–25]. However, the contribution of MHC alleles versus host genetic background has not been examined during chronic Cl-13 infection.

In the present study, we sought to determine the genetic influences on CD8 T cell exhaustion and the development of persistent viral infection using MHC congenic inbred and first generation (F1) crosses of various inbred mouse strains. Our results indicate that increasing MHC diversity allows for a broader CD8 T cell response that is able to more rapidly control persistent LCMV Cl-13 infection. However, F1 mice expressing a H-2^bxd^ haplotype on different genetic backgrounds exhibit differences in both CD8 T cell exhaustion and CD4 T cell subset distribution, resulting in differences in viral control and disease. Unexpectedly, H-2^bxd^ mice on a C57BL background succumb to LCMV Cl-13 infection in a T-cell- and IFN-γ-dependent manner. Thus, both MHC haplotype and genetic background together influence host resistance to CD8 T cell exhaustion and disease susceptibility following LCMV Cl-13 infection.

## Results

### Host genetics influences viral persistence and Cl-13-induced mortality

**Fig 1:**
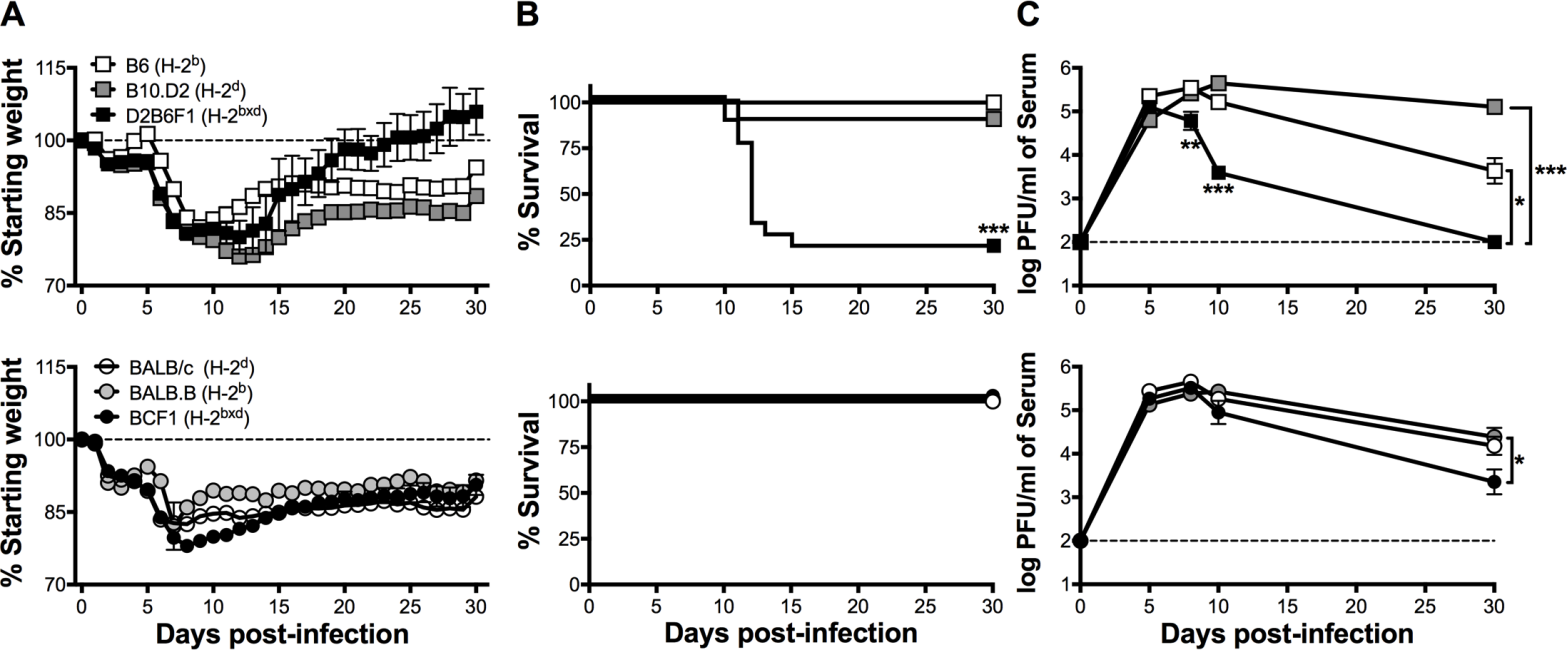
H-2^bxd^ mice on a C57BL background control virus but succumb to Cl-13 infection. Mice were infected i.v. with LCMV Cl-13. (A) Weight loss and (B) survival were monitored following infection. (C) Viral titers were determined by plaque assay at the indicated time points in the serum following infection. Data depict cumulative data from 2–4 independent experiments *(n* = 7–18). Survival statistics were determined by Mantel-Cox Log-rank test. Titer differences were determined by t test at each time point. *, *p* < 0.05; **, *p* < 0.01; ***, *p* < 0.001.

Here we sought to determine the impact of host genetics on the course of a chronic LCMV Cl-13 infection. Mice expressing an H-2^b^, H-2^d^ or H-2^bxd^ haplotype on either a C57BL or BALB background were examined. MHC H-2^bxd^ haplotype mice on the C57BL background were generated via an F1 cross of C57BL/6 and B10.D2 (D2B6F1) mice. Using a similar strategy H-2^bxd^ haplotype mice on a BALB background were created via an F1 cross of BALB/c and BALB.B (BCF1) mice. To determine susceptibility to chronic infection, mice were infected with LCMV Cl-13 and disease and viral clearance were monitored. Whereas all strains developed weight loss following infection, only D2B6F1 mice exhibited mortality (Fig 1A and 1B). D2B6F1 mice also displayed early control of viral replication and mice that survived exhibited enhanced viral clearance in both serum and systemic organs (Fig 1C, upper panel and S1 Fig). In addition, BCF1 mice exhibited enhanced viral clearance at day 30 post-infection as compared to MHC congenic mice on the same background (Fig 1C, lower panel and S1 Fig). Notably, viral tropism did not differ between mouse strains as determined by intracellular expression of the viral nucleoprotein (NP) in macrophages and various DC subsets in the spleen (S2 Fig). These results indicate that H-2^bxd^ haplotype mice mediate more rapid viral control than either of their H-2^d^ or H-2^b^ homozygous controls. However, on the C57BL genetic background, D2B6F1 mice control viral replication more rapidly but also succumb to infection, indicating a combined role for both MHC-linked genes and non-MHC background genes in the mortality resulting from LCMV Cl-13 infection.

### H-2^bxd^ haplotype mice prioritize H-2^d^-restricted CD8 T cell responses over H-2^b^-restricted responses following acute LCMV infection

**Fig 2:**
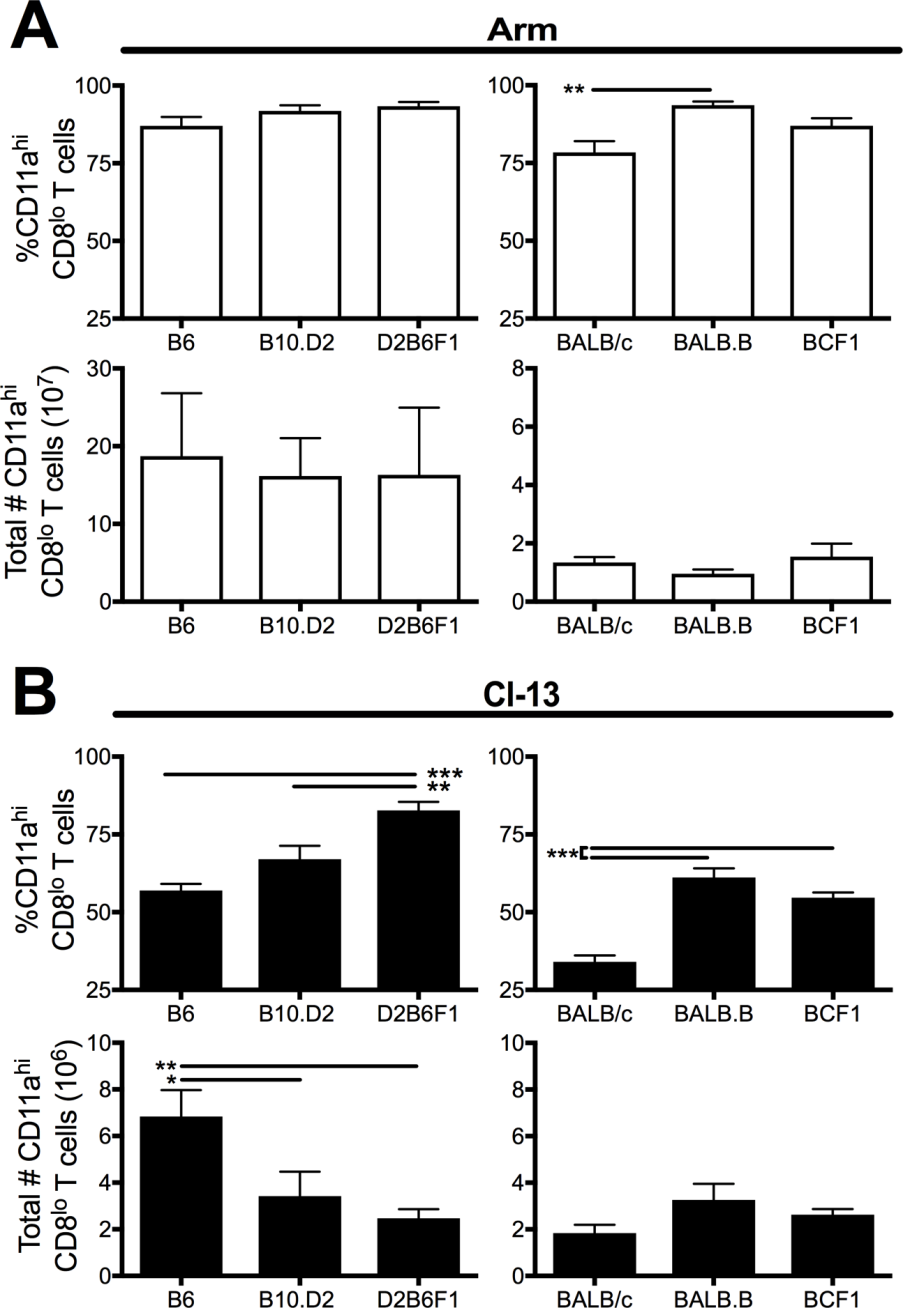
D2B6F1 mice induce the largest frequency of activated CD8 T cells following Cl-13 infection. Mice were infected with either LCMV Arm i.p. or Cl-13 i.v. and spleens were harvest 8 days later. The frequency (top) and total number (bottom) of CD11a^hi^CD8^lo^ T cells are shown following (A) Arm and (B) Cl-13 infections. Data depict cumulative results from 3–4 independent experiments (*n* = 8–17). Statistics were determined by one-way ANOVA with Tukey’s multiple comparison test. *, *p* < 0.05; **, *p* < 0.01; ***, *p* < 0.001.

**Fig 3:**
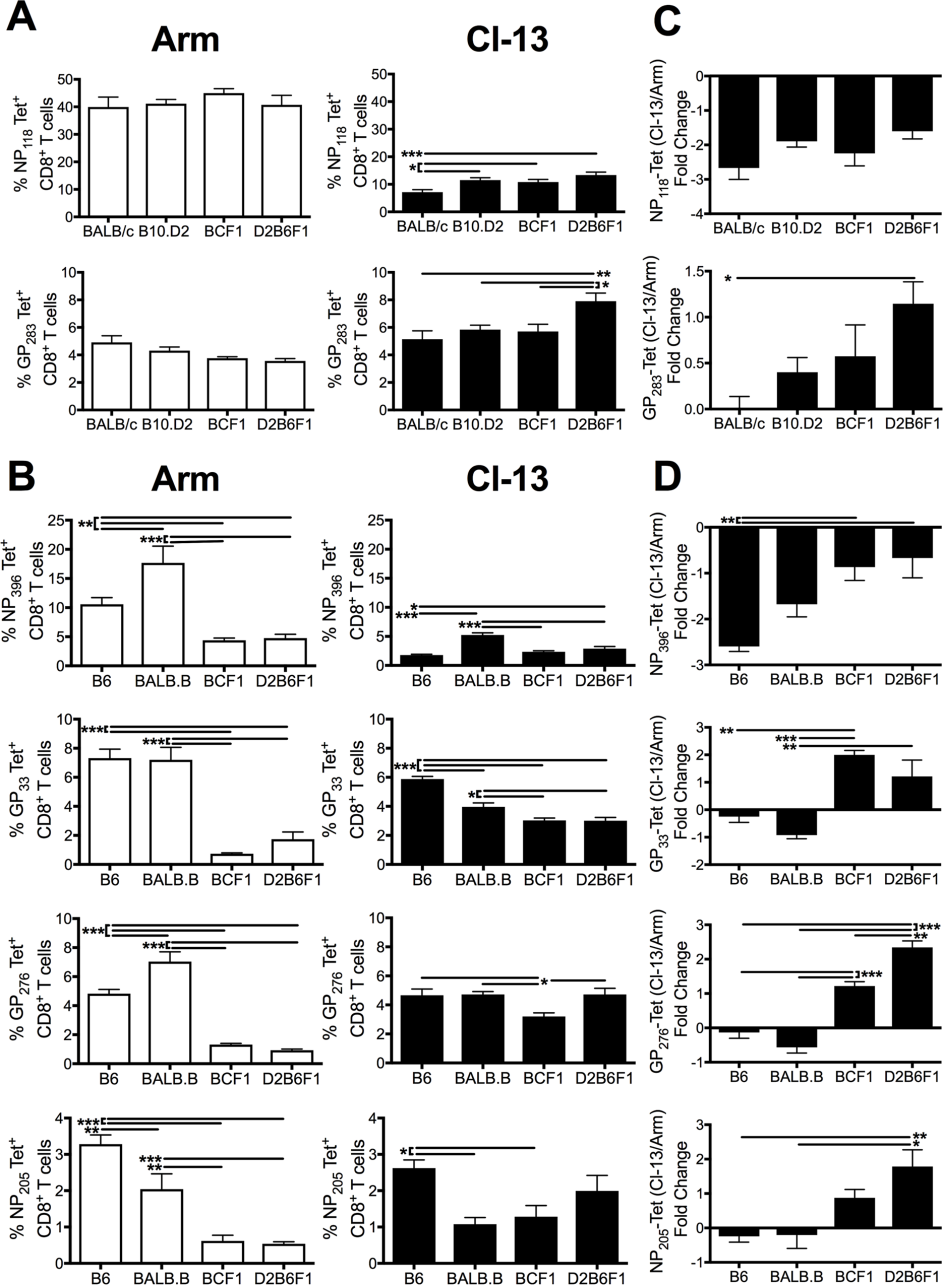
H-2^bxd^ haplotype mice prioritize H-2^d^-restricted responses. Mice were infected with either LCMV Arm i.p. or Cl-13 i.v. and spleens were harvest 8 days later. The frequency of tetramer-specific CD8 T cells were identified for (A) H-2^d^- and (B) H-2^b^- specific epitopes following Arm (left) and Cl-13 (right) infection. The fold change in the frequency of tetramer^+^ from Cl-13 compared to Arm infection was examined for (C) H-2^d^- and (D) H-2^b^-specific epitopes. Data depict cumulative results from 3–4 independent experiments (*n* = 8–17). Statistics were determined by one-way ANOVA with Tukey’s multiple comparison test. *, *p* < 0.05; **, *p* < 0.01; ***, *p* < 0.001.

To determine the magnitude and epitope hierarchy of the CD8 T cell response induced in MHC H-2^bxd^ haplotype mice under ideal T cell priming conditions, the CD8 T cell response was measured 8 days after an acute LCMV-Arm infection (Fig 2, white bars). The total number and frequency of virus-specific CD8 T cells, as measured by expression of the surrogate activation marker CD11a and reduced expression of CD8, did not differ between heterozygous (F1) and homozygous MHC haplotype inbred control mice following acute LCMV infection (Fig 2A). Further examination of epitope-specific responses revealed maintenance of both the immunodominant NP_118_ and subdominant GP_283_ H-2^d^-restricted responses in the MHC H-2^bxd^ haplotype mice compared to homozygous MHC controls (Fig 3A, S3A Fig; white bars). However, within H-2^b^-restricted epitopes, responses to the immunodominant epitopes NP_396_, GP_33_, and GP_276_ as well as the subdominant epitope NP_205_ were significantly reduced in F1 mice compared to inbred controls (Fig 3B, S3B Fig; white bars). These data indicate that MHC H-2^bxd^ haplotype mice induce a T cell response of similar magnitude as homozygous control mice by prioritizing H-2^d^-specific CD8 T cell responses over H-2^b^-specific responses following acute LCMV infection. Thus, following an acute LCMV infection NP_118_ represents the immunodominant epitope and all of the H-2^b^-specific epitopes become subdominant.

### D2B6F1 mice maintain the highest frequency of functional CD8 T cells during chronic viral infection

While the relative epitope-specific distribution within the virus-specific CD8 T cell response was altered in H-2^bxd^ haplotype mice following acute infection, the magnitude of the overall virus-specific CD8 T cell response as determined by the number of CD11a^hi^CD8^lo^ T cells was not altered (Fig 2A). However, during chronic viral infection, D2B6F1 mice exhibited an increased frequency of CD11a^hi^CD8^lo^ T cells (Fig 2B). This indicates that a higher proportion of CD8 T cells were responding to Cl-13 infection in D2B6F1 mice than the inbred control mice.

Previous studies have shown that in B6 mice the immunodominace hierarchy is altered between LCMV Arm and Cl-13 infection [13, 26]. NP_396_ represents the largest immunodominant epitope following Arm infection. However, NP_396_-specific CD8 T cells are the most rapid to exhaust and be deleted following Cl-13 infection [13, 26]. In contrast, the frequency of CD8 T cells specific to subdominant epitopes are maintained allowing for the emergence of subdominant epitope-specific CD8 T cells during chronic Cl-13 infection [13, 26]. Examination of individual CD8 T cell epitopes following chronic Cl-13 infection revealed that D2B6F1 mice either maintained or increased the frequency of CD8 T cells responding to many of the epitopes regardless of the proportion of CD8 T cells that responded during acute Arm infection (Fig 3A and 3B; black bars). Moreover, both BCF1 and D2B6F1 mice maintained a higher proportion of antigen-specific CD8 T cells than inbred mice following chronic versus acute infection (Fig 3C and 3D). In the F1 mice, a larger proportion of the H-2^b^-epitopes were expressed during chronic Cl-13 infection compared to during acute Arm infection, potentially due to their altered immunodominance hierarchy within the total CD8 T cell response (Fig 3C and 3D). Thus, by broadening the number of epitopes recognized by the CD8 T cells in MHC heterozygous mice, we observe an increase in the total frequency of CD8 T cells responding based on CD11a^hi^CD8^lo^ expression (Fig 2B). Taken together, our results indicate that increasing MHC heterogeneity results in an increase in the frequency of responding CD8 T cells during a chronic viral infection.

### Impact of host genetic background on the effector functions of CD8 T cells during chronic infection

**Fig 4:**
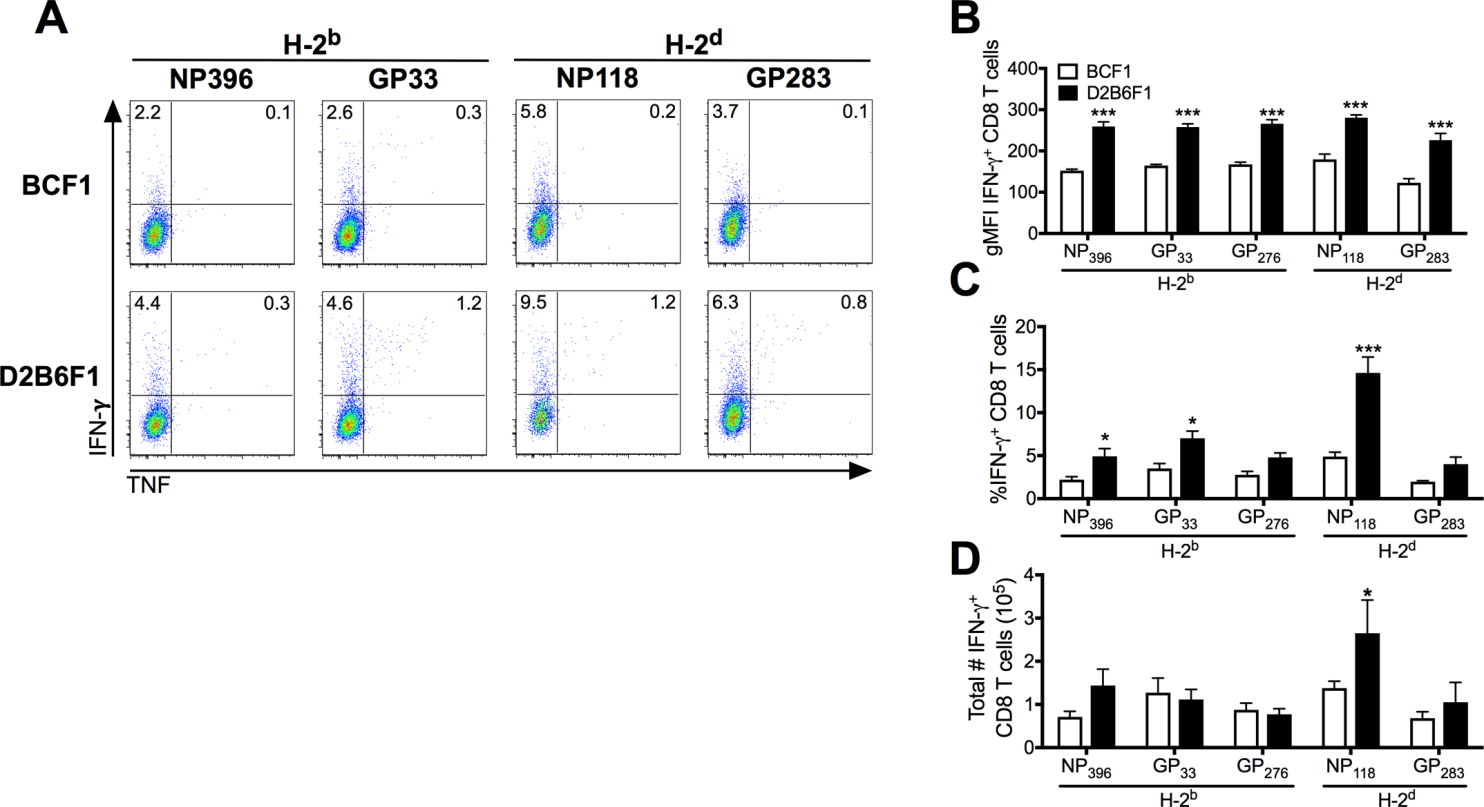
C57BL background H-2^bxd^ mice exhibit increased CD8 T cell effector functions compared to BALB background mice. Mice were infected with LCMV Cl-13 i.v and spleens were harvested 8 days later. (A) The frequency of IFN-γ^+^ (y-axis) and TNF^+^ (x-axis) CD90.2^+^ CD8^+^ T cells are shown following peptide stimulation. Geometric MFI (B), cumulative frequencies (C) and total numbers (D) of IFN-γ^+^ CD8 T cells are shown. Data depict cumulative results from 3–4 independent experiments (*n* = 8–16). Statistics were determined by two-way ANOVA with Sidak’s multiple comparison test. *, *p* < 0.05; ***, *p* < 0.001.

H-2^bxd^, D2B6F1 mice on the C57BL genetic background were the only strain tested that succumbed to Cl-13 infection (Fig 1C). D2B6F1 mice also mounted a higher frequency CD8 T cell response than mice on the BALB/c background as determined by the frequency of cells that upregulate CD11a and downregulate CD8 (Fig 2B). Previous studies have shown that CD8 T cell exhaustion early following Cl-13 infection prevents CD8 T-cell-induced mortality [27, 28]. An increase in the frequency of responding CD8 T cells in D2B6F1 mice suggests that CD8 T cells may play a role in mediating the Cl-13-induced mortality observed in this strain. Therefore, we sought to determine the functional capacity of the CD8 T cells and the degree of CD8 T cell exhaustion. Both H-2^b^- and H-2^d^-restricted CD8 T cell epitopes from D2B6F1 mice exhibited a greater frequency of IFN-γ and IFN-γ/TNF co-producing CD8 T cells than BCF1 mice following either Arm or Cl-13 infection (Fig 4A and 4C; S4 Fig). Interestingly, approximately ~70% of the CD8 T cell response in D2B6F1 mice is directed against NP_118_. In addition, the total number of NP_118_-specific CD8 T cells, representing the largest of the CD8 T cell epitopes, was significantly increased in D2B6F1 compared to BCF1 mice following Cl-13 infection (p < 0.05; Fig 4D). This result indicates that in addition to an increase in the frequency of responding CD8 T cells, D2B6F1 mice retain more functional virus-specific CD8 T cells than BCF1 mice. This further suggests a role for non-MHC host genetic background genes in the induction of the CD8 T cell response and the resulting Cl-13-induced mortality in D2B6F1 mice.

### CD8 T cell exhaustion is influenced by the genetic background of the host

**Fig 5:**
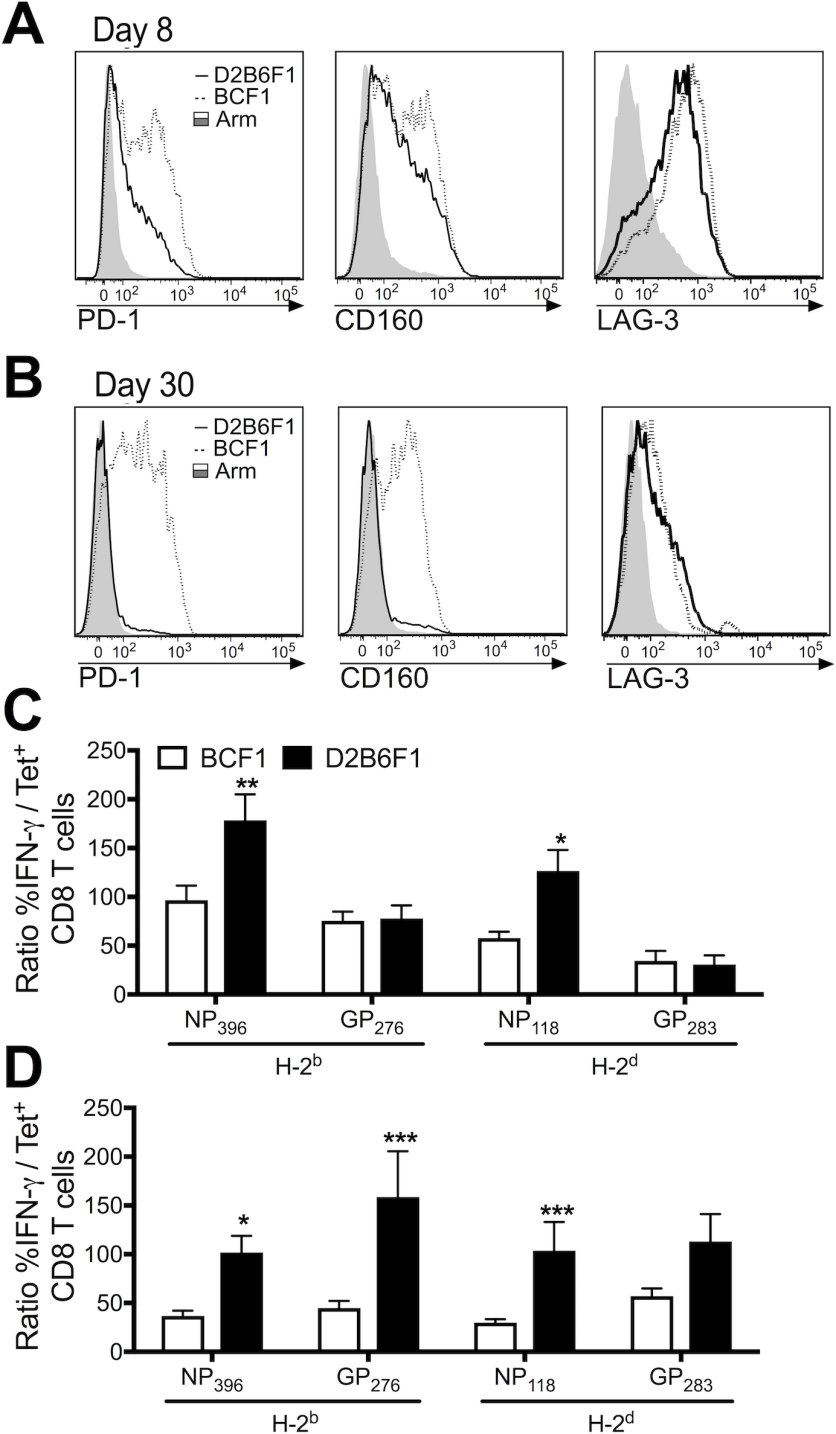
C57BL background mice exhibit reduced CD8 T cell exhaustion compared to BALB background mice. PD-1, CD160, and LAG-3 expression from day 8 (A) and day 30 (B) CD11a^hi^CD8^lo^ T cells from Cl-13-infected BCF1 (dotted line), D2B6F1 (solid line), and Arm-infected (shaded) mice. The ratio of IFN-γ expressing CD8 T cells divided by the frequency of tetramer^+^ CD8 T cells from day 8 (C) and day 30 (D) Cl-13-infected BCF1 (white) and D2B6F1 mice (black). Data depict cumulative results from 3–6 independent experiments (*n* = 6–22). Statistics were determined by two-way ANOVA with Sidak’s multiple comparison test. *, *p* < 0.05; **, *p* < 0.01; **, *p* < 0.001.

CD8 T cell exhaustion has been shown to occur in a stepwise fashion, where CD8 T cells initially lose the ability to produce TNF, followed by IFN-γ and eventually resulting in CD8 T cell deletion [13, 29]. In addition, exhausted CD8 T cells upregulate expression of multiple inhibitory receptors such as PD-1, CD160 and LAG-3 [27, 30]. To determine the extent of CD8 T cell exhaustion in D2B6F1 and BCF1 mice, we examined PD-1, CD160 and LAG-3 expression and epitope-specific IFN-γ production during Cl-13 infection. As early as day 8 post-infection, D2B6F1 mice exhibited reduced PD-1 and CD160 expression on their CD8 T cells (Fig 5A). However, LAG-3 expression was similar between strains (Fig 5A). By day 30 post-infection, virus was cleared from both LCMV Arm-infected mice and Cl-13-infected D2B6F1 mice and PD-1 and CD160 were no longer expressed on the CD8 T cells (Fig 5B). However, Cl-13-infected BCF1 mice maintained high levels of virus and inhibitory ligand expression on their CD8 T cells at this time point (Fig 5B). This difference in inhibitory ligand expression highlights a major difference between the MHC H-2^bxd^ haplotype response on the C57BL and BALB background. At both day 8 (Fig 5C) and day 30 (Fig 5D) post-infection, D2B6F1 mice expressed a higher frequency of functional CD8 T cells than BCF1 mice. Thus, CD8 T cells from D2B6F1 mice exhibited reduced CD8 T cell exhaustion compared to their BCF1 counterparts. This increased CD8 T cell functionality may contribute to both the rapid viral control but also the Cl-13-mediated mortality observed in D2B6F1 mice.

### D2B6F1 mice exhibit an increased CD4 T cell response during chronic viral infection

**Fig 6:**
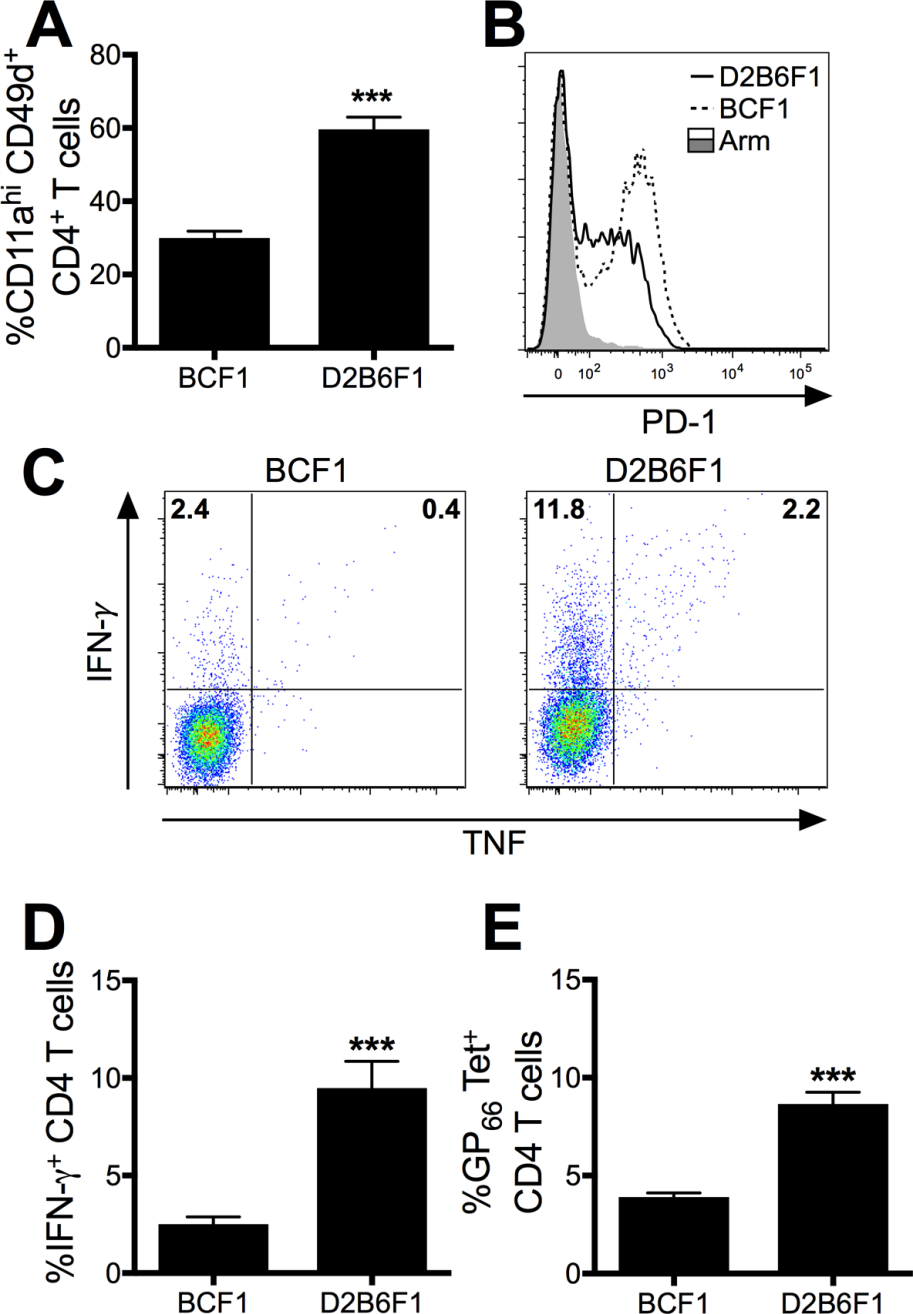
The C57BL background induces a robust CD4 T cell response during chronic viral infection. Mice were infected with LCMV Cl-13 i.v and spleens were harvested 8 days later. (A) The frequency of CD11a^hi^CD49d^+^ CD4 T cells following infection. (B) PD-1 expression in Cl-13-infected BCF1 (dotted line), D2B6F1 (solid line) mice and Arm-infected mice (filled line). (C) Flow plots and (D) cumulative data of IFN-γ^+^ and TNF^+^ cells following GP_61_ peptide stimulation. (E) The frequency of GP_66_-tetramer^+^ CD4 T cells. Data depict cumulative results from 3–5 independent experiments (*n* = 12–21). Statistics were determined by *t* test. ***, *p* < 0.001.

CD4 T cells have been shown to play a vital role in limiting CD8 T cell exhaustion during chronic viral infection in B6 mice [11, 31]. To determine if the CD4 T cell response contributes to the reduced CD8 T cell exhaustion observed in D2B6F1 mice, we next examined the frequency and function of the virus-specific CD4 T cells. During LCMV Cl-13 infection, D2B6F1 mice exhibited a significantly increased frequency of antigen-specific CD4 T cells as measured by the surrogate activation markers CD11a and CD49d [32], as compared to CD4 T cells from BCF1 mice (p<0.0001; Fig 6A). This increased frequency of virus-specific CD4 T cells coincides with a reduced expression of the exhaustion marker PD-1 on the responding CD4 T cells (Fig 6B). D2B6F1 mice also exhibited an increased frequency of GP_61_-specific CD4 T cells by both IFN-γ production and GP_66_ tetramer staining (Fig 6C–6E). Our results indicate that expression of the MHC H-2^bxd^ haplotype on the C57BL background results in the induction of a larger frequency of CD4 T cells following Cl-13 infection than is elicited in mice expressing the H-2^bxd^ haplotype on the BALB background.

### H-2^bxd^ haplotype mice on the C57BL genetic background maintain Th1-polarized CD4 T cells during chronic viral infection

**Fig 7:**
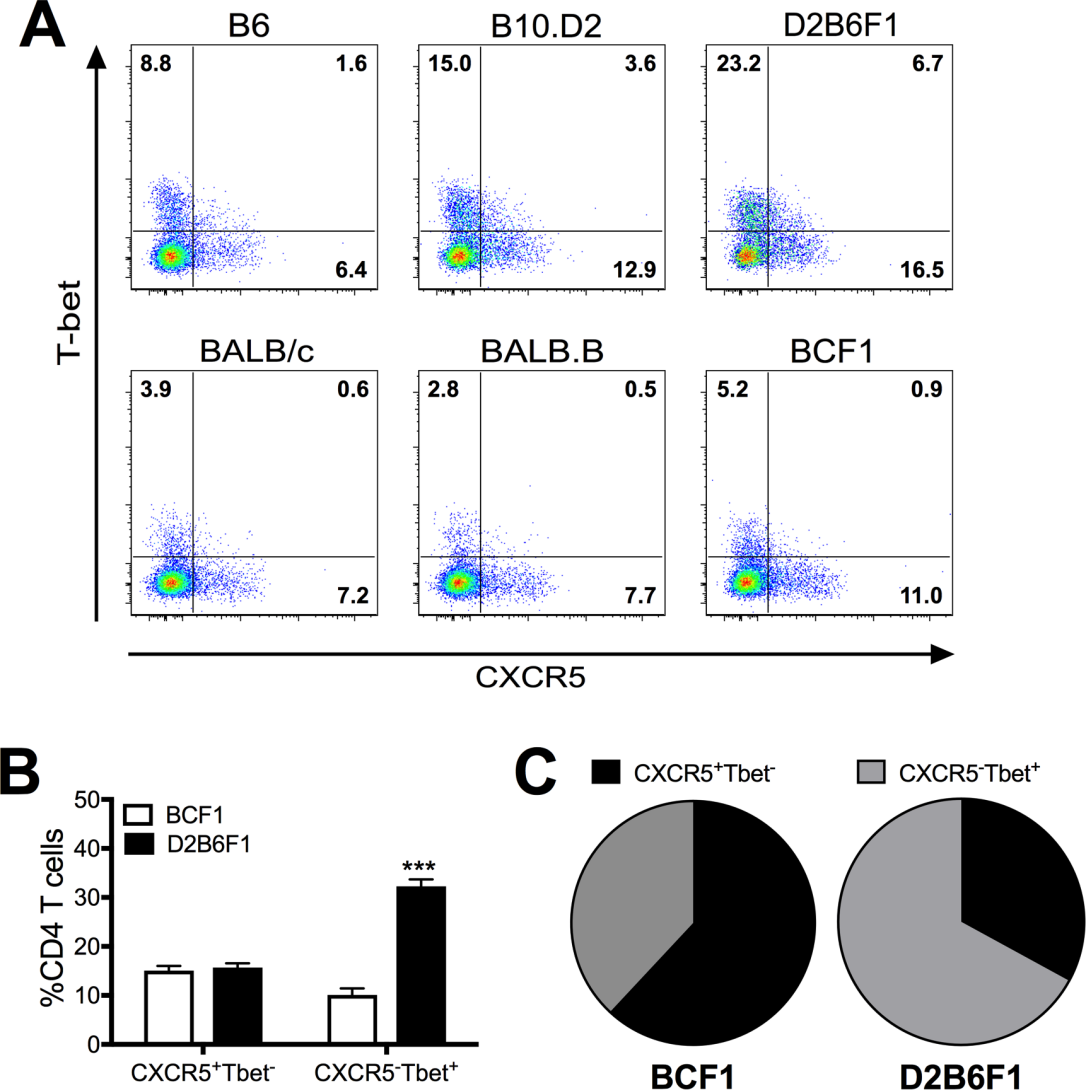
Cl-13 infection of mice on a C57BL background results in an enhanced Th1 response. Mice were infected with LCMV Cl-13 i.v and spleens were harvested 8 days post-infection. (A) Flow plots depicting Tbet and CXCR5 expression on CD4 T cells 8 days post-infection. (B) Cumulative frequencies of CXCR5 and Tbet expressing CD4 T cells following Cl-13 infection. (C) Pie charts depict the proportions of CXCR5 and Tbet expressing CD4 T cells following Cl-13 infection. Data depict cumulative results from 3–5 independent experiments (*n* = 5–21). Statistics were determined by two-way ANOVA with Sidak’s multiple comparison test. ***, *p* < 0.001.

Following a chronic Cl-13 infection, CD4 T cells have been shown to convert from Th1 to a Tfh phenotype [18]. In an acute LCMV Arm infection, the Th1 cells are maintained and IFN-γ production has been shown to play a pivotal role in supporting CD8 T cell effector activity [18, 33]. Furthermore, Th1 cells contribute to viral clearance during an acute infection [34]. In a chronic infection, IL-21 production by Tfh cells has been shown to be the primary mechanism by which CD4 T cells help maintain CD8 T cell effector functions at late stages of infection [18, 35, 36]. Here we find that D2B6F1 mice, which exhibit reduced CD8 T cell exhaustion and more rapid viral clearance, maintain a higher proportion of Th1 (Tbet^+^ CD4^+^) cells while BCF1 mice exhibit more Tfh (CXCR5^+^ CD4^+^) cells (Fig 7). Thus, our results indicate that maintaining Th1-biased CD4 T cell responses may prevent CD8 T cell exhaustion and viral persistence early during Cl-13 infection allowing for more rapid viral control.

### CD8 and CD4 T cells contribute to Cl-13-induced mortality in D2B6F1 mice

**Fig 8:**
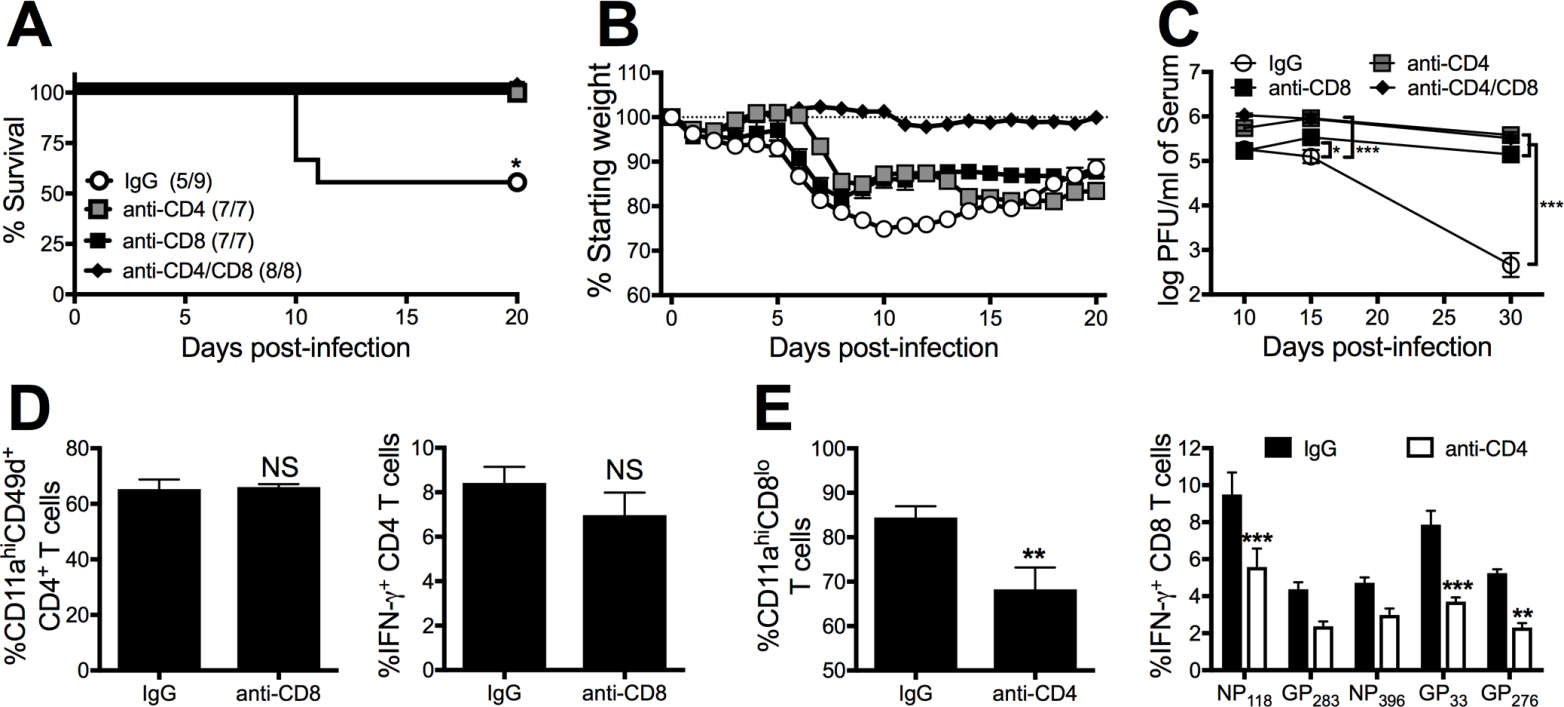
Both CD4 and CD8 T cells contribute to Cl-13-induced mortality in D2B6F1 mice. D2B6F1 mice were treated with 200 μg IgG, anti-CD4, anti-CD8 or both on day -2, 2, 6, 10 following LCMV Cl-13 infection. (A) Survival (survived/infected) and (B) weight loss were monitored following infection. (C) Serum viral titers were determined on day 10 post-infection. (D) The frequency of CD11a^hi^CD49^+^ and GP_61_-peptide specific CD4 T cells following stimulation were assessed on day 8 post-infection in the PBL. (E) The frequency of CD11a^hi^CD8^lo^ and peptide-specific CD8 T cells following stimulation were assessed on day 8 post-infection in the PBL. Data depict cumulative results from 2 independent experiments (*n* = 5–9). Statistics were determined by *t* test, one-way ANOVA with Tukey’s post-test, or two-way ANOVA with Sidak’s multiple comparison test. Survival statistics were determined by log rank Mantel-Cox test compared to IgG treated mice. *, *p* < 0.05; **, *p* < 0.01; ***, *p* < 0.001.

CD8 T cell exhaustion occurs in order to prevent CD8 T-cell-mediated immunopathology [27, 28]. In the absence of inhibitory receptors during the early stages of chronic viral infection, CD8 T cells have been shown to both clear virus and induce lethal immunopathology [27, 28]. During LCMV Cl-13 infection, CD4 T cells provide vital help for CD8 T-cell-mediated viral control [11, 31]. To determine if the increased effector functions observed for both CD8 and CD4 T cells in D2B6F1 mice contributes to their mortality, we depleted CD8 and CD4 T cells prior to Cl-13 infection. In the absence of either CD4 or CD8 T cells, D2B6F1 mice survived Cl-13 infection (Fig 8A and 8B). In addition, CD8 T cell depletion did not alter the frequency of the CD4 T cell response, indicating that CD4 T cells alone cannot induce mortality in the absence of CD8 T cells (Fig 8D). In contrast, CD4 T cell depletion resulted in a significant reduction in the frequency of activated and epitope-specific CD8 T cells (Fig 8E). This indicates that CD4 depletion may enhance the survival of D2B6F1 mice by affecting the magnitude of the CD8 T cell response. Furthermore, CD8 and/or CD4 T cell depletion resulted in increased viral titers, confirming their critical role in the enhanced viral control observed in D2B6F1 mice (Fig 8C). These results indicate that the increased CD8 T cell response observed in D2B6F1 mice results in both enhanced viral control and mortality in D2B6F1 mice.

### IFN-γ contributes to mortality and viral control in D2B6F1 mice

**Fig 9:**
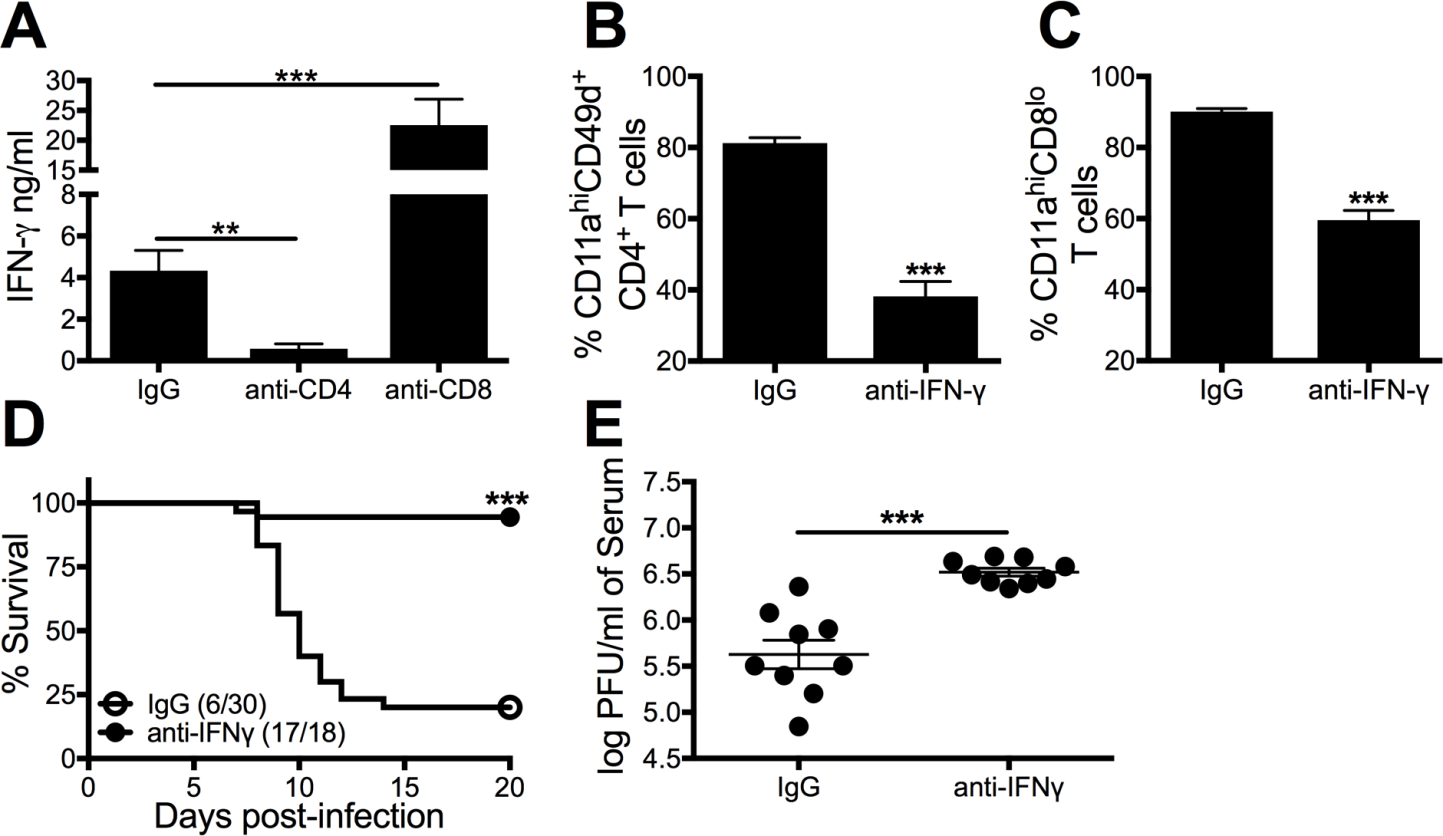
IFN-γ contributes to mortality and viral control following Cl-13 infection in D2B6F1 mice. (A) D2B6F1 mice were treated with 200 μg IgG, anti-CD4, anti-CD8 or both on day -2, and 2 following LCMV Cl-13 infection and serum was collected on day 6 for IFN-γ ELISA. In the remaining panels D2B6F1 mice were treated with 200 μg IgG or anti-IFN-γ on day -1, 1, 3, 5, 7, and 9 days post-Cl-13 infection. The frequency of activated CD4 (B) and CD8 (C) T cells were analyzed on day 8 post-infection. (D) Survival (survived/infected) was monitored following infection. (E) Serum viral titers were measured on day 10 post-infection. Data depict cumulative results from 2–5 independent experiments (*n* = 7–30). Survival statistics were determined by log rank Mantel-Cox test compared to IgG treated mice. One-way ANOVA and Tukey’s post-test were used to determined titer statistics. **, *p* < 0.01; ***, *p* < 0.001.

To determine the role of Th1-associated cytokines on Cl-13-induced mortality in D2B6F1 mice, IFN-γ was depleted prior to infection. IFN-γ is produced by both CD8 and CD4 T cells during chronic viral infection and has been shown to contribute to viral clearance and immunopathology [37–39]. Depletion of CD4 T cells, but not CD8 T cells, resulted in a significant decrease in the amount of IFN-γ protein in the serum during Cl-13 infection (p<0.001; Fig 9A). In addition, IFN-γ neutralization reduced the magnitude of both the CD4 and CD8 T cell response in D2B6F1 mice (Fig 9B and 9C). Importantly, IFN-γ neutralization resulted in increased survival (Fig 9D). Furthermore, IFN-γ neutralization resulted in increased viral titers in the serum (Fig 9E). These results indicate that CD4 T-cell-mediated IFN-γ production plays a critical role in CD8 T cell induction, viral control and the mortality observed in D2B6F1 mice during Cl-13 infection.

## Discussion

Host susceptibility to the establishment of a persistent viral infection is linked to the ability of the host to mount an effective CD8 T cell response to control virus replication. However, CD8 T-cell-mediated viral control is limited by T cell exhaustion during chronic infection, which occurs to prevent T-cell-mediated pathology [28]. Thus, understanding the mechanisms that induce an effective CD8 T cell response to prevent viral persistence without the induction of immunopathology is vital to the prevention of chronic infection. Here we show both MHC heterogeneity and host genetic background significantly impact CD8 T cell exhaustion and viral clearance.

CD8 T cells are important for clearing acute LCMV Arm infection and help to maintain viral control during chronic LCMV Cl-13 infection [11, 40]. Increasing the diversity of viral antigens presented during chronic viral infection often increases viral control [2, 5, 6]. We show that both D2B6F1 mice and BCF1 mice induce an increased frequency of activated CD8 T cells compared to their inbred controls resulting in enhanced viral control. This is similar to the enhanced viral control observed in HLA heterozygote individuals during both HCV and HIV infection [2, 6]. In addition, D2B6F1 mice induce both a larger CD8 T cell response and maintain increased effector functions compared to BCF1 mice. Similar to CD8 T cell depletion during SIV infection of macaques [41], CD8 T cell depletion in D2B6F1 mice ameliorates the enhanced viral control observed during Cl-13 infection. Thus, individuals that induce a CD8 T cell response that recognizes a broader range of epitopes are more capable of controlling and clearing chronic viral infections.

Increasing CD8 T cell function is desired for enhanced control and clearance of chronic viral infection. However, the purpose of CD8 T cell exhaustion is to prevent CD8 T-cell-mediated immunopathology. Previous studies have shown that PD-1 deficiency early during infection results in CD8 T-cell-mediated mortality [27, 28]. This is due to an increase in CD8 T cell numbers and function in PD-1-deficient mice (22, 23). Similarly, we observe the mortality in D2B6F1 mice is associated with an increased CD8 T cell response and reduced CD8 T cell exhaustion. In the absence of PD-1, mice succumb to chronic infection due to perforin-mediated killing of endothelial cells by CD8 T cells resulting in increased vascular permeability [28]. However, D2B6F1 mice do exhibit low levels of PD-1 expression potentially resulting in a less severe and delayed mortality compared to PD-1-deficient mice.

Increasing MHC diversity resulted in CD8 T-cell-mediated mortality that was dependent on the host genetic background. Increasing MHC diversity allows for induction of a broader CD8 T cell response. However, during chronic viral infection T cell exhaustion limits the size and function of the virus-specific T cell response [13, 29]. Our results indicate that the genetic background of the host influences the extent of CD8 T cell exhaustion. CD4 T cells play an important role in CD8 T cell exhaustion [11, 31] and mouse strain susceptibility to infection often differs based on their ability to mount an appropriate CD4 T cell response [24, 42–44]. During acute infection, a Th1-biased response is induced to aid in rapid viral clearance [24, 34, 42–45]. However, BALB/c mice often induce weaker Th1 responses than B6 mice [42, 43]. For example, BALB/c mice are more susceptible to *Leishmania* and RSV infections due to their inability to mount robust Th1 responses [23, 24, 43]. Thus, B6 mice are able to more easily clear Th1-dependent pathogens [42]. In addition, perforin-deficient mice succumb to acute LCMV Arm infection in a IFN-γ-dependent manner [46]. However, this effect is only observed in B6 mice, not BALB/c mice [47]. This difference is believed to be due to a weaker IFN-γ response in BALB/c mice, and only when memory CD8 cells are generated prior to LCMV infection do perforin-deficient BALB/c mice succumb to IFN-γ-mediated death [47]. While both BALB/c and B6 mice are susceptible to persistent viral infection, only H-2^bxd^ expressing mice on the C57BL background succumb to Cl-13 infection. This is not due to differences in viral tropism between mouse strains as determined by intracellular expression of the LCMV NP protein in macrophages and various dendritic cell subsets. The mortality exhibited by the D2B6F1 mice may be due to the relative distribution of CD4 Th subsets. At the peak of the T cell response, D2B6F1 mice were found to induce a larger Th1 response than BCF1 mice. This increased Th1 response in D2B6F1 mice may help to mediate the larger and more functional CD8 T cell response observed in D2B6F1 mice versus BCF1 mice. Thus, D2B6F1 mice exhibit the combined effect of enhanced CD4 T cell help due to increased Th1 polarization and reduced viral load and antigen as a result of a broader T cell response because of the increase in MHC heterogeneity resulting in reduced CD8 T cell exhaustion as compared to the other mouse strains examined. Therefore, both the immune environment and the increased MHC diversity contribute to the CD8 T-cell-dependent viral clearance and mortality observed in D2B6F1 mice.

The majority of studies examining CD8 T cell exhaustion following LCMV Cl-13 infection are conducted in inbred B6 mice. However, our findings demonstrate that the host genetic background can profoundly influence the outcome of an LCMV Cl-13 infection. Thus, utilization of hosts with more diverse genetic backgrounds are likely to yield additional factors involved in CD8 T cell exhaustion and the establishment of chronic infections opening new potential avenues for therapeutic interventions to prevent chronic infection in humans.

## Materials and methods

### Mice and infection

BALB/c and C57BL/6 mice were purchased from National Cancer Institute (Fredrick, MD) and bred in the University of Iowa animal facility. BALB.B and B10.D2 mice were purchased from the Jackson Laboratory (Fredrick, MD) and bred in the University of Iowa animal facility. First generation BALB.B x BALB/c (BCF1) mice and B10.D2 x C57BL/6 (D2B6F1) mice were created in the University of Iowa animal facility. All mice were utilized between 6–8 weeks of age. The Arm and Cl-13 strains of LCMV were gifts from Dr. Raymond Welsh (University of Massachusetts Medical School, Worcester, MA) and propagated in BHK-21 cells (ATCC). For LCMV Arm infection, mice were infected i.p. with 2 x 10^5^ PFU. For LCMV Cl-13 infection, mice were infected i.v. with 1.8 x 10^6^ PFU. Disease was assessed daily by monitoring weight loss and survival.

### Ethics statement

All experimental procedures utilizing mice were approved by the University of Iowa Animal Care and Use Committee. The experiments were performed under strict accordance to the Office of Laboratory Animal Welfare guidelines and the PHS Policy on Humane Care and Use of Laboratory Animals.

### Plaque assays

Serum, lung, liver, and kidneys were harvested from Cl-13-infected mice. Serum was isolated from whole blood collected in heparinized tubes (Fisher; Pittsburgh, PA), capped (Lecia Microsystems; Richmond, IL) and spun at 2500 rpm for 30 min. Serum was collected and snap-frozen in liquid nitrogen prior to storage at -80°C. Lung, liver and kidneys were homogenized and processed for plaque assays as previously described [48].

### Antibody depletion

For T cell depletion, mice were administered 200 μg of rat IgG (MP Biomedicals, Aurora, OH) or anti-CD4 (clone GK1.5) and/or anti-CD8 (clone 2.43) i.p. on days -2, 2, 6, 10 relative to infection. Depletion was verified in the blood on day 6 post-infection. For cytokine depletion, mice were administered 200 μg of rat IgG (MP Biomedicals), anti-IFN-γ (clone XMG1.2), or anti-TNF (clone XT22) i.p. on days -1, 1, 3, 5, 7, and 9 relative to infection.

### Flow cytometry analysis

Cells were isolated from the blood and spleen as previously described [48]. For detection of antigen-specific cytokine responses cells were stimulated with 1μM NP_396-404_, GP_33-41_, GP_276-286_, NP_118-126_, GP_283-291_, or GP_61-80_ (Biosynthesis, Lewisville, TX) in the presence of 10 μg/ml BFA (Sigma Aldrich) for 5 hour at 37°C in 10% FCS-supplemented RPMI. For tetramer staining, cells were incubated with a single tetramer (NP_118-126_, GP_283-291_, GP_66-77_, and NP_205-212_ from NIH Tetramer Core Facility; NP_396-404_, GP_33-41_, and GP_276-286_ made in house) for 30 min at 4°C for CD8 tetramers or 90 min at 37°C for the CD4 tetramer. Following tetramer staining or stimulation, cells were fixed using 1-step Fix/Lyse Solution (eBioscience, San Diego, CA). For staining of transcription factors, cells were fixed with Foxp3 staining buffer set (eBioscience) and stained intracellularly for Tbet (clone 4B10). Cells were stained with the extracellular mAbs specific to CD90.2 (clone 53–2.1), CD8α (clone 53–6.7), CD4 (clone RM4-5), CD11a (clone M17/4), CD49d (clone R1-2), and PD-1 (clone RMP1-30, all previous antibodies from eBioscience and Biolegend) for 15 min at 4°C. For CXCR5 staining, cells were stained with the mAb for CXCR5 (biotin, BD Pharmingen) at room temperature for 30 min followed by streptavidin-PE (eBioscience) in FACS buffer. Cells were stained for the intracellular mAbs specific to IFN-γ (XMG1.2), TNF (clone MP6-XT22) in FACS Buffer containing 0.5% saponin (Sigma-Aldrich) for 30 min at 4°C. Intracellular staining for LCMV NP using a directly PE-conjugated Rat anti-LCMV NP mAb clone VL-4 [49] (a gift from Dirk Homann, Icahn School of Medicine at Mount Sinai, New York, NY) was performed as previously described [50]. Samples were run on a BD LSRFortessa flow cytometer and data was analyzed using FlowJo software (Tree Star Inc, Ashland, OR).

### Statistical analysis

Graphical and statistical analyses were performed using Prism software (GraphPad Software Inc., San Diego, CA) with error bars representing the SEM. *P* values were considered significant when *p*<0.05.

## Supporting information

S1 Fig H-2^bxd^ mice control chronic viral infection more rapidly than inbred mice. Mice were infected i.v. with LCMV Cl-13 and viral titers were determined by plaque assay at day 30 following infection. Data depict cumulative results from 4 independent experiments (*n* = 3–15). Titer differences were determined by one-way ANOVA with Tukey’s post-test. *, *p* < 0.05; **, *p* < 0.01; ***, *p* < 0.001.
(TIFF)

S2 Fig Viral tropism does not vary between mouse backgrounds. Mice were infected i.v. with LCMV Cl-13 and viral tropism was determined by the frequency (A, C) and total number (B, D) of NP^+^ DCs and Macrophages on day 8 post-infection. All cell populations were negative for expression CD3, CD19, and DX5 and positive for CD45. cDCs were also gated on CD11c^+^ F4/80^-^. pDCs were gated on CD11c^+^ F4/80^-^ B220^+^. Macrophages were F4/80^+^. Statistics were determined by two-way ANOVA with Sidak’s multiple comparison test.
(TIFF)

S3 Fig The total numbers of Tet-specific CD8 T cells. Mice were infected with either LCMV Arm i.p. or Cl-13 i.v. and spleens were harvest 8 days later. The total number of tetramer-specific CD8 T cells were identified for (A) H-2^d^- and (B) H-2^b^- specific epitopes following Arm (left) and Cl-13 (right) infection. Data depict cumulative results from 3–4 independent experiments (*n* = 8–17). Statistics were determined by one-way ANOVA with Tukey’s multiple comparison test. *, *p* < 0.05; **, *p* < 0.01; ***, *p* < 0.001.
(TIFF)

S4 Fig CD8 T cell cytokine production is impaired following Cl-13 infection compared to Arm infection. Mice were infected with either LCMV Arm i.p. or Cl-13 i.v. and spleens were harvest 8 days later. Flow plots depicting cytokine producing CD8 T cells following NP118 (A) or NP396 (B) stimulation are shown. Cumulative frequencies are shown for IFN-γ^+^TNF^+^ CD8 T cells following Arm (C) and Cl-13 (D) infection. Data depict cumulative results from 3–4 independent experiments (*n* = 8–17). Statistics were determined by two-way ANOVA with Sidak’s multiple comparison test. *, *p* < 0.05; ***, *p* < 0.001.
(TIFF)
