# Supplementary figures and images for: Host genetics play a critical role in controlling CD8 T cell function and lethal immunopathology during chronic viral infection
Source: PLoS Pathog. 2017 Jul 17;13(7):e1006498. doi: 10.1371/journal.ppat.1006498 (PMC5531689; doi:10.1371/journal.ppat.1006498)

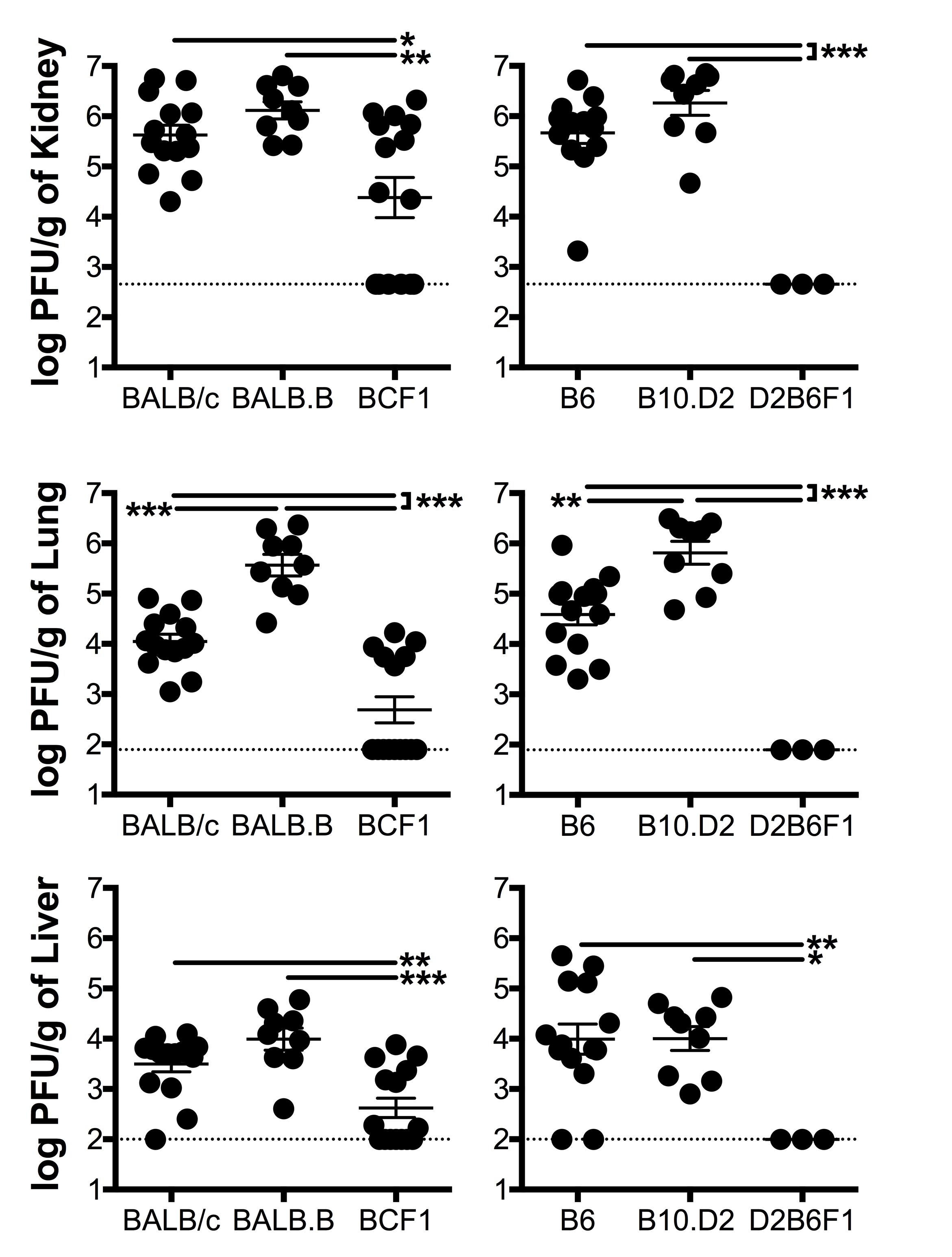

Supplement: S1 Fig — Mice were infected i.v. with LCMV Cl-13 and viral titers were determined by plaque assay at day 30 following infection. Data depict cumulative results from 4 independent experiments (n = 3–15). Titer differences were determined by one-way ANOVA with Tukey’s post-test. *, p < 0.05; **, p < 0.01; ***, p < 0.001. (TIFF) [file ppat.1006498.s001.tiff]

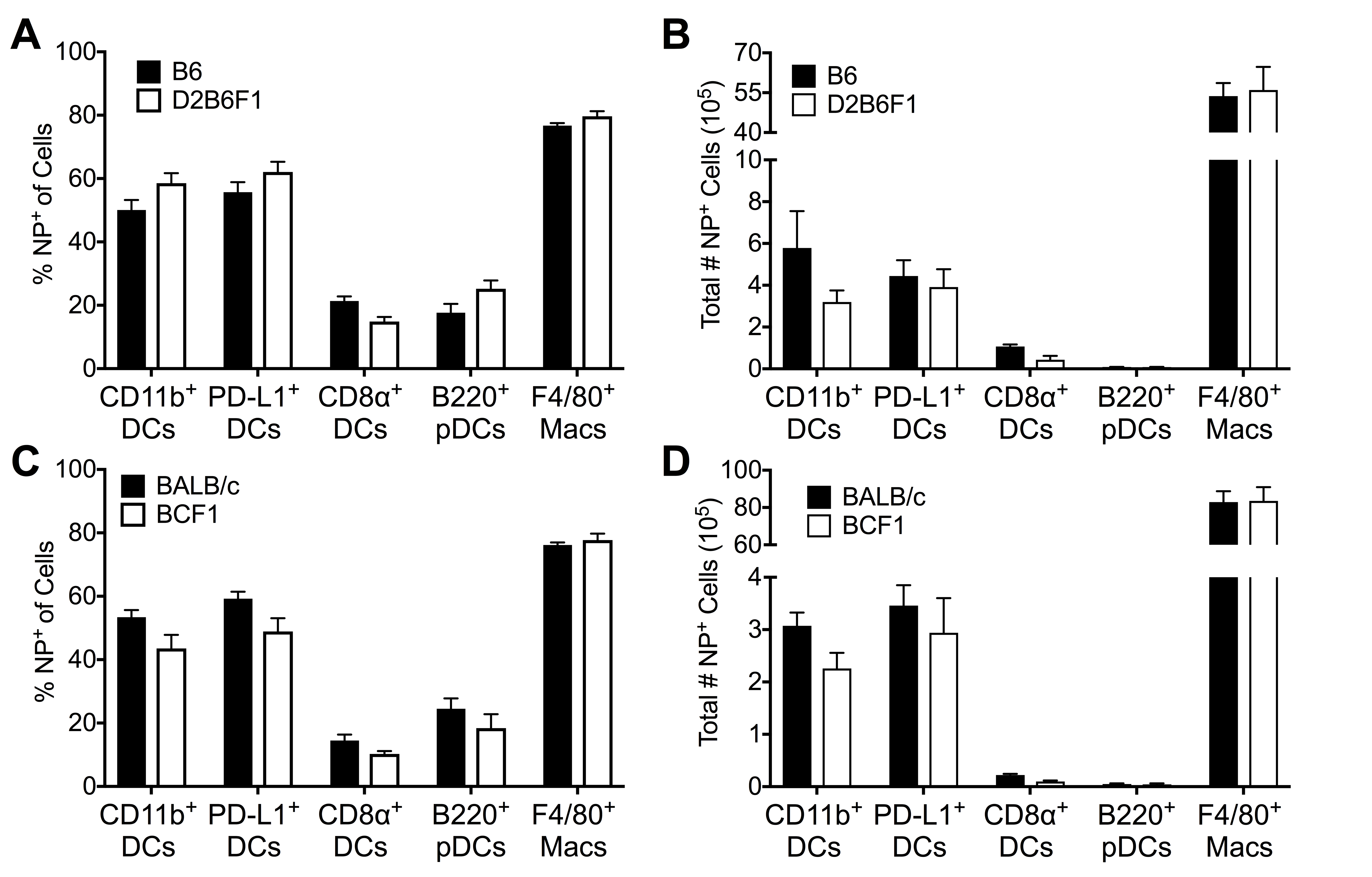

Supplement: S2 Fig — Mice were infected i.v. with LCMV Cl-13 and viral tropism was determined by the frequency (A, C) and total number (B, D) of NP+ DCs and Macrophages on day 8 post-infection. All cell populations were negative for expression CD3, CD19, and DX5 and positive for CD45. cDCs were also gated on CD11c+ F4/80-. pDCs were gated on CD11c+ F4/80- B220+. Macrophages were F4/80+. Statistics were determined by two-way ANOVA with Sidak’s multiple comparison test. (TIFF) [file ppat.1006498.s002.tiff]

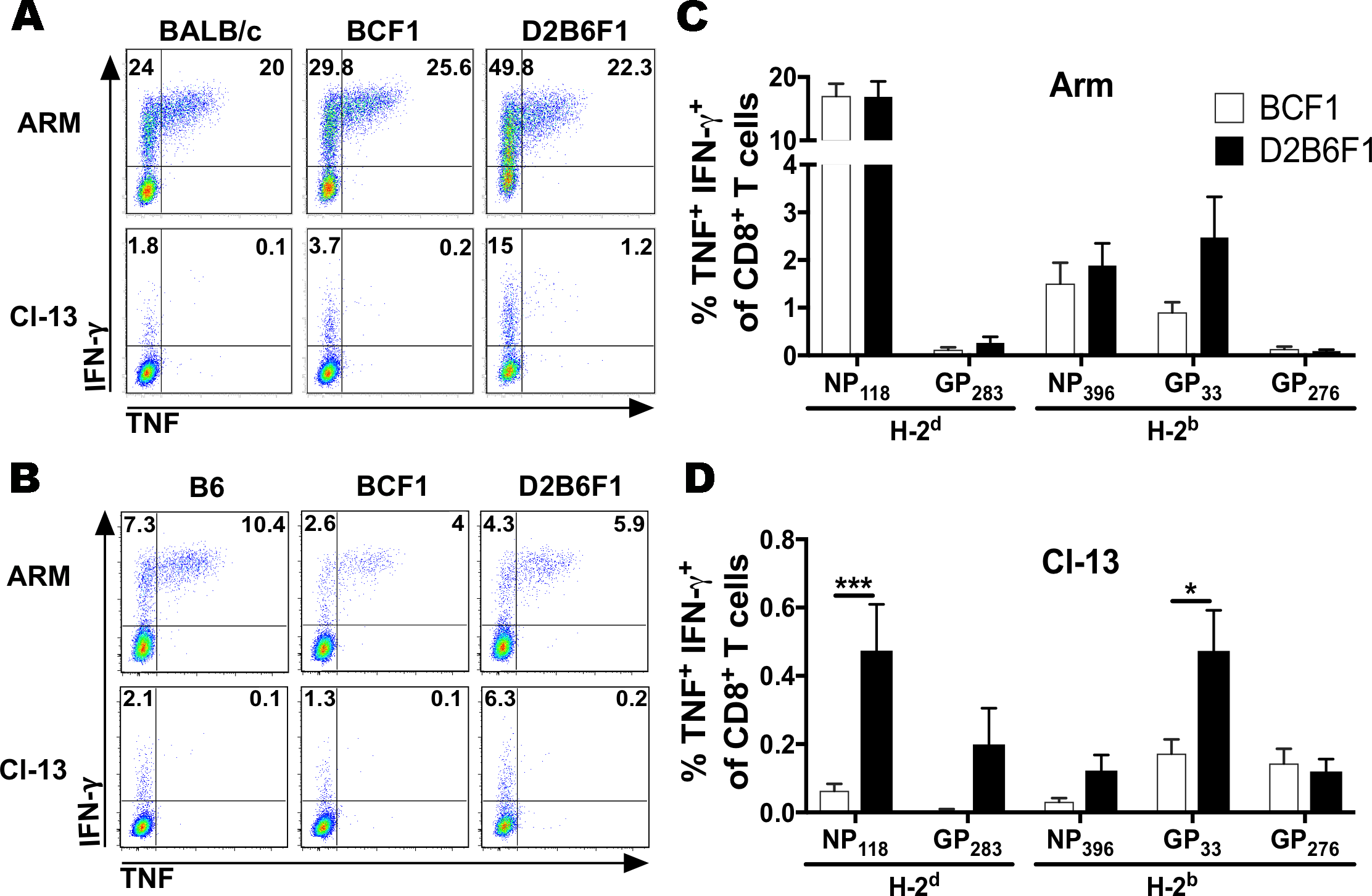

Supplement: S3 Fig — Mice were infected with either LCMV Arm i.p. or Cl-13 i.v. and spleens were harvest 8 days later. The total number of tetramer-specific CD8 T cells were identified for (A) H-2d- and (B) H-2b- specific epitopes following Arm (left) and Cl-13 (right) infection. Data depict cumulative results from 3–4 independent experiments (n = 8–17). Statistics were determined by one-way ANOVA with Tukey’s multiple comparison test. *, p < 0.05; **, p < 0.01; ***, p < 0.001. (TIFF) [file ppat.1006498.s003.tiff]

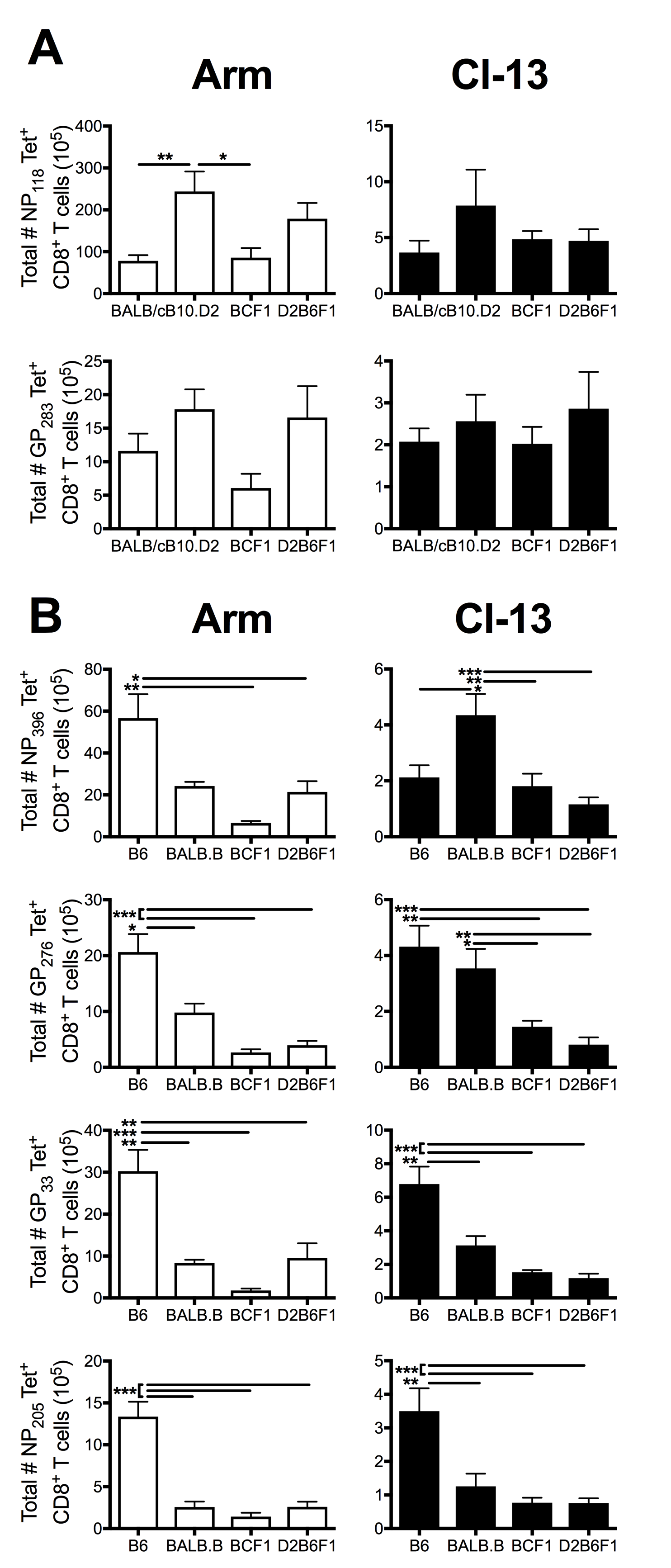

Supplement: S4 Fig — Mice were infected with either LCMV Arm i.p. or Cl-13 i.v. and spleens were harvest 8 days later. Flow plots depicting cytokine producing CD8 T cells following NP118 (A) or NP396 (B) stimulation are shown. Cumulative frequencies are shown for IFN-γ+TNF+ CD8 T cells following Arm (C) and Cl-13 (D) infection. Data depict cumulative results from 3–4 independent experiments (n = 8–17). Statistics were determined by two-way ANOVA with Sidak’s multiple comparison test. *, p < 0.05; ***, p < 0.001. (TIFF) [file ppat.1006498.s004.tiff]
